# Regulation of Angiogenesis Discriminates Tissue Resident MSCs from Effective and Defective Osteogenic Environments †

**DOI:** 10.3390/jcm9061628

**Published:** 2020-05-28

**Authors:** R. J. Cuthbert, E. Jones, C. Sanjurjo-Rodríguez, A. Lotfy, P. Ganguly, S. M. Churchman, P. Kastana, H. B. Tan, D. McGonagle, E. Papadimitriou, P. V. Giannoudis

**Affiliations:** 1 Leeds Institute of Rheumatic and Musculoskeletal Disease, University of Leeds, Leeds LS16 7PS, UK; R.J.Cuthbert@leeds.ac.uk (R.J.C.); E.Jones@leeds.ac.uk (E.J.); clara.sanjurjo@udc.es (C.S.-R.); umpga@leeds.ac.uk (P.G.); S.M.Churchman@leeds.ac.uk (S.M.C.); hiangboon@hotmail.com (H.B.T.); D.G.McGonagle@leeds.ac.uk (D.M.); 2 Department of Biomedical Sciences, Medicine and Physiotherapy, University of A Coruña, CIBER-BBN-Institute of Biomedical Research of A Coruña (INIBIC), A Coruña 15001, Spain; 3 Biotechnology and Life Sciences Department, Faculty of Postgraduate Studies for Advanced Sciences (PSAS), Beni-Suef University, Beni-Suef 62511, Egypt; lotfy_bio@hotmail.com; 4 Department of Pharmacy, School of Health Sciences, University of Patras, Patras 265 04, Greece; kastana.penny@gmail.com (P.K.); epapad@upatras.gr (E.P.); 5 NIHR Leeds Biomedical Research Center, Chapel Allerton Hospital, Leeds LS7 4SA, UK

**Keywords:** MSCs, fracture healing, non-union, induced periosteum, osteogenesis, regenerative medicine

## Abstract

Background: The biological mechanisms that contribute to atrophic long bone non-union are poorly understood. Multipotential mesenchymal stromal cells (MSCs) are key contributors to bone formation and are recognised as important mediators of blood vessel formation. This study examines the role of MSCs in tissue formation at the site of atrophic non-union. Materials and Methods: Tissue and MSCs from non-union sites (*n* = 20) and induced periosteal (IP) membrane formed following the Masquelet bone reconstruction technique (*n* = 15) or bone marrow (*n* = 8) were compared. MSC content, differentiation, and influence on angiogenesis were measured in vitro. Cell content and vasculature measurements were performed by flow cytometry and histology, and gene expression was measured by quantitative polymerase chain reaction (qPCR). Results: MSCs from non-union sites had comparable differentiation potential to bone marrow MSCs. Compared with induced periosteum, non-union tissue contained similar proportion of colony-forming cells, but a greater proportion of pericytes (*p* = 0.036), and endothelial cells (*p* = 0.016) and blood vessels were more numerous (*p* = 0.001) with smaller luminal diameter (*p* = 0.046). MSCs showed marked differences in angiogenic transcripts depending on the source, and those from induced periosteum, but not non-union tissue, inhibited early stages of in vitro angiogenesis. Conclusions: In vitro, non-union site derived MSCs have no impairment of differentiation capacity, but they differ from IP-derived MSCs in mediating angiogenesis. Local MSCs may thus be strongly implicated in the formation of the immature vascular network at the non-union site. Attention should be given to their angiogenic support profile when selecting MSCs for regenerative therapy.

## 1. Introduction

Non-union (NU) occurs in approximately 10% of long bone fractures, with an incidence of approximately 19 per 100,000 population per annum in the developed world, and is associated with considerable morbidity, expenditure, and social cost [1,2]. NU can be broadly classified into either hypertrophic or atrophic based on characteristic radiological features [3,4]; while it is widely accepted that inadequate mechanical fixation is the primary contributing factor to the development of hypertrophic NU [5,6,7], the causes of atrophic NU are less well understood.

The probability that any fracture will develop into atrophic NU is dependent on multiple factors including loss of bone at the fracture site, infection, extensive soft tissue damage, and open fractures [8,9]. Patient specific factors such as age; gender; the administration of non-steroidal anti-inflammatory drugs; smoking; and medical comorbidities including anaemia, diabetes, and hormone disorders as well as genetic factors have also been suggested to contribute to NU risk [8,9]. Despite these observations, reliable predictive measures of those at risk of NU are not available and the nature of the underlying biological mechanisms is poorly understood.

Mesenchymal stromal cells (MSCs) are widely accepted as being key contributors to new bone formation following fracture, and in surgery are often taken from bone marrow (BM) and used as components of autologous grafts [10,11]. Indeed, the use of autologous MSCs for the treatment of NU is well established and widely practiced [10,12,13,14,15]. Recently, there has also been a great deal of interest in the ability of MSCs to act as regenerative agents independently of their ability to directly form new tissue [16,17,18,19]. Several studies in the field of ischaemic heart injury have investigated their ability to act as mediators of angiogenesis by the release of trophic factors, including vascular endothelial growth factor (VEGF) [19,20,21]. The relationship between MSCs and blood vessels has been the subject of much interest, and some have suggested that all MSCs are in fact pericytes [22,23,24]. Regardless of the answer to this question, it is clear that MSCs are closely associated with blood vessels and, therefore, interactions between MSCs and endothelial cells may be of crucial importance in physiological healing.

Historically, it was suggested that vascularity of the NU site was a key difference between hypertrophic and atrophic NU, with some suggesting atrophic NUs to be avascular and essentially inert [25]. However, this pre-conception has been challenged by several publications which found significant vascularity in atrophic tissue [25,26,27]. An alternative hypothesis could be that atrophic NU tissue lacks the required regenerative cells, or that regenerative cells are present but lack potency. This was supported by the work of Bajada et al., who confirmed that although MSCs are present at the site of atrophic NU, they had reduced osteogenic potential and increased cell senescence compared to those isolated from BM [26]. However, it must be noted that MSCs in the bone marrow may be fundamentally different from those in peripheral tissues, not least in their well described role in maintaining the haematopoietic niche [28,29,30], a property which has not been demonstrated for MSCs resident in other tissues.

The Masquelet technique is a form of guided bone regeneration used for the repair of large bone defects [31,32]. In the first stage, a membrane is formed following the implantation of a cement spacer. The cement is subsequently removed and replaced with autologous bone graft, leaving the membrane in place [33]. Karger et al. reported healing defects larger than 10 cm using this technique and union was achieved in an average of nine months with a success rate of 94% [34]. Moreover, time to union was not dependent on defect size [34]; this is important given that defects of up to 25 cm have been repaired using this technique [35].

These impressive results have generated interest in the properties of the membrane formed; indeed, it has been shown to possess osteogenic, osteoinductive, and angiogenic properties [33,36,37]. We recently reported characteristics very similar to periosteum in terms of thickness, cellularity, and anatomical location [38], leading us to coin the phrase “induced periosteum” (IP). Additionally, IP contained a far greater frequency of MSC than reported in BM aspirates [38,39,40,41], and MSCs isolated from the IP were functionally equivalent to those isolated from matched normal periosteum, a well-accepted source of MSCs with osteogenic potential comparable to those isolated from bone marrow [42,43,44,45]. To date, no studies have addressed the fundamental difference in MSC function between atrophic NU tissue and tissue from a site of high osteogenic potential. In this study, we examine the cellular content, histological characteristics, and vascularity of NU and IP tissue, as well as the angiogenic potential of MSCs isolated from these tissues. Multipotent differentiation potential of NU MSCs was compared with the standard BM MSCs.

## 2. Materials and Methods

### 2.1. Patient Recruitment

This work was performed at Leeds Institute of Rheumatic and Musculoskeletal Disease, University of Leeds, and Department of Pharmacy, School of Health Sciences, University of Patras. Study participants were patients admitted for treatment of atrophic NU fractures, critical size defects using the induced membrane technique, or elective orthopaedic surgery to either the upper or lower extremity. All patients gave informed consent and research was carried out in compliance with the Helsinki Declaration. Ethical approval was obtained from the U.K. Health Research Authority, Leeds East Research Ethics Committee for the harvesting of these tissues.

In total, 20 patients suffering from fracture NU (median age: 53, range 23–81, 11 males: 9 female) and 15 patients treated for critical size defects using the induced membrane technique (median age 61, range 19–80, 10 male: 5 female) were recruited. Bone marrow (BM) aspirates were collected from eight patients undergoing elective orthopaedic surgery (median age 38, range 19–52, 3 males: 5 female).

### 2.2. Tissue Collection and Processing

Fibrotic NU tissue was collected directly from the NU site. At the time of surgery, following exposure of the fracture site, the fibrotic tissue lying directly between the fractured bone fragments was excised and collected for study.

Samples for the IP were collected at a mean time of 7.6 weeks (range 6–9) from the first stage of the technique (insertion of cement spacer). Approximately 1 cm of membrane tissue was harvested from the center of the bone defect area. Tissue was either digested by incubation in collagenase solution to generate a single cell suspension for culture initiation or flow cytometry analysis, or processed for histology [38]. A subset of samples tissue was bisected for both enzymatic digestion and processing for histology. BM aspirates were seeded directly in culture in StemMACS media (Miltenyi Biotec, Bisley, Surrey, UK) with penicillin and streptomycin (Thermo Fisher Scientific, Paisley, UK) to generate BM MSC cultures, as previously described [38].

### 2.3. Colony Forming Potential

A colony forming unit fibroblast (CFU-F) assay was used to assess MSC content in enzymatically digested NU and IP tissues [38]. A total of 1 × 10^4^ cells were seeded in duplicate into 10 cm diameter tissue culture plates (Corning B.V. Life Sciences, Amsterdam, The Netherlands) and incubated in StemMACS media with penicillin and streptomycin in standard culture conditions for 14 days, followed by fixation in 4% formalin solution (Sigma Aldrich, Gillingham, Dorset, UK) and staining with methylene blue (Sigma Aldrich). Colonies were defined as ≥50 tightly clustered cells. BM aspirates were seeded volumetrically directly in culture in StemMACS media, as previously described [39].

### 2.4. In Vitro Osteogenesis, Chondrogenesis, and Adipogenesis

Enzymatically released cells from the NU tissue were incubated in standard culture conditions in StemMACS media. After 48 h, the culture media was renewed and expansion continued for 14 days with twice weekly half media changes. Subsequently, cells were trypsinised and re-seeded at a density of 4500 cells/cm^2^ and expansion continued for a maximum of two passages. For these experiments, BM MSCs were expanded similarly and used as control. Both types of cultures were seeded into differentiation assays at the same seeding density, as previously described [38]. Measurement of alkaline phosphatase activity was assessed at 14 days after the initiation of osteogenic culture conditions, as previously described [46,47]. The degree of mineralisation, by measurement of calcium, was carried out at 21 days post initiation. For chondrogenic assays, ChondroPrime SF media (PAA laboratories, GE Healthcare, Chicago, IL, USA) was used, over a period of 21 days [46,47]; cell pellets were visualised after staining with Toludine blue solution [38] using an Eclipse E1000 light microscope (Nikon, Kinston upon Thames, Surrey, UK). For adipogenic assays, cells were seeded into NH adipoDiff medium (Miltenyi Biotec) and cultured, over 21 days [46,47], followed by staining with oil red O (Sigma) as previously described [38]. Images were captured using a CKX41 inverted light microscope (Olympus, Southend on Sea, UK).

### 2.5. Histology and Immunohistochemistry

Histology and immunohistochemistry (IHC) were carried out on paraformaldehyde-fixed histological IP and NU tissue sections (*n* = 8 and 9 respectively). Haematoxylin and eosin staining was performed according to standard protocols. IHC staining was carried out using EnVision peroxidase/DAB+ detection system (Dako, Stockport, UK) and specific anti-human antibodies (Supplementary Table S1). In cases where antigen retrieval was required, this was performed by heating sections in boiling 10 nM citrate buffer (pH 6.0) in a 900 W microwave oven for 10 min. All images were captured using an Eclipse E1000 light microscope (Nikon). For measurement of average blood vessel size, up to six photomicrographs (dependent on tissue size) were taken at 200× magnification following CD31 labelling. Measurement of blood vessel areas was then performed blinded using ImageJ free access image processing and analysis software available from http://rsb.info.nih.gov/ij/. The number of vessels was counted in at least 20 different random fields of each experimental group, as previously described [48]. Analysis of CD45, stromal-derived factor 1 (SDF1), vascular endothelial growth factor 1 (VEGF), and bone morphogenetic protein 2 (BMP-2) expression was performed using NIS elements BR (Nikon Instruments Inc., Melville, USA ) image analysis software.

### 2.6. Flow Cytometry

Enzymatically digested single cell suspensions were incubated with specific fluorescently labelled primary antibodies for 30 min at 4 °C at manufacturer recommended concentrations (Supplementary Table S1). Cells were then washed and incubated with 4’,6-diamidino-2-phenylindole to assess viability, and then analysed using a LSR II flow cytometer (BD) and FacsDiva analysis software (BD, Oxford, UK). The proportions of pericytes of (CD45-CD34-CD146+) [49], endothelial cells of (CD45-CD31+) [40], and lymphocytes of (CD45+ low side-scatter) [40] were measured.

### 2.7. Quantitative Real-Time PCR (qPCR)

A 48-gene array was employed, focusing predominantly on markers of angiogenesis as well as common MSC lineage (chondro-, adipo-, osteo) markers [50]; for a full list of genes, see Supplementary Table S2. IP and NU derived MSCs (*n* = 7 and 8, respectively) were cultured up to passage 2, before being trypsinised and washed. Control unmatched, expanded (p2) iliac crest BM MSCs (*n* = 8) and skin fibroblast cell lines (*n* = 3, Lonza and ATCC) were also prepared. Cells were lysed and RNA isolated using Norgen Total RNA Isolation Kit (Geneflow, Lichfield, UK). cDNA was produced using High Capacity cDNA reverse transcription kit for use on the Taqman low density array (TLDA) with 2× gene expression mix (all Thermo Fisher) [50]. Further individual Taqman assays were used to analyse additional transcripts and for validation of the TLDA. All gene expression analysis was using the 2^−ΔCt^ method, normalised to *HPRT*. Greater than two-fold expression change was considered different, when significant.

### 2.8. In Vitro Angiogenesis

Matrigel-based angiotube formation assay was performed to assess the paracrine angiogenic potential of IP and NU MSCs. MSCs originating from IP and NU tissue were seeded in cell culture flasks (Corning Inc., New York, NY, USA) and incubated under standard conditions in MSC growth media (StemMACS Media). At 80% confluence, supernatant was removed and new MSC growth media was added, as previously described [51]. Following a further 72 h incubation, MSC conditioned media was centrifuged at 300× *g* for 10 min to eliminate dead cells and debris, and the supernatant was then frozen at −80 °C until use. The MSCs from each flask were trypsinized and counted to allow standardization on a per cell basis.

Then, 2 × 10^4^ human umbilical vein endothelial cells (HUVECs; Lonza, Basilea, Switzerland) were seeded on Matrigel (Corning Inc.) pre-coated 96-well culture plates (Corning Inc.). Endothelial cells were incubated in MSC conditioned media, HUVEC medium (endothelial cell growth medium 2; PromoCell, Heildelberg, Germany) was used as a positive control and unconditioned MSC growth medium was used to assess baseline angiogenesis. All conditions were assessed in duplicate and two images per well were taken after 3 h, using a Cytation 5 imaging plate reader (BioTek, Winooski, VT, USA).

Images were analyzed using the macro Angiogenic Analyzer for ImageJ software. We compared the results from the two cell types, as described by Khoo et al. [52], by calculating the number of junctions, number of tubules, and tubule length. Data obtained were normalized to 1 × 10^6^ cells and expressed as percentage of change fold, compared with unconditioned MSC growth medium. Supplementary Table S3 summarises control tissues used throughout the study.

### 2.9. Statistical Analysis

The Mann-Whitney U test was used to determine the statistical significance for all comparisons between BM and NU and IP and NU samples. SPSS version 21 (IBM, New York, NY, USA) was used for statistical analysis throughout, while GraphPad Prism version 7.02 (GraphPad Software Inc., La Jolla, CA, USA) was used to generate all graphs. All quantitative measurements were performed in triplicate (technical replicates) and the biological replicates (donor numbers) are specified in each figure legend. Box plots displayed as median (line), interquartile range (box) and extreme values (whiskers).

## 3. Results

### 3.1. Colony Formation and Differentiation Capacity of Resident MSCs

**Figure 1:**
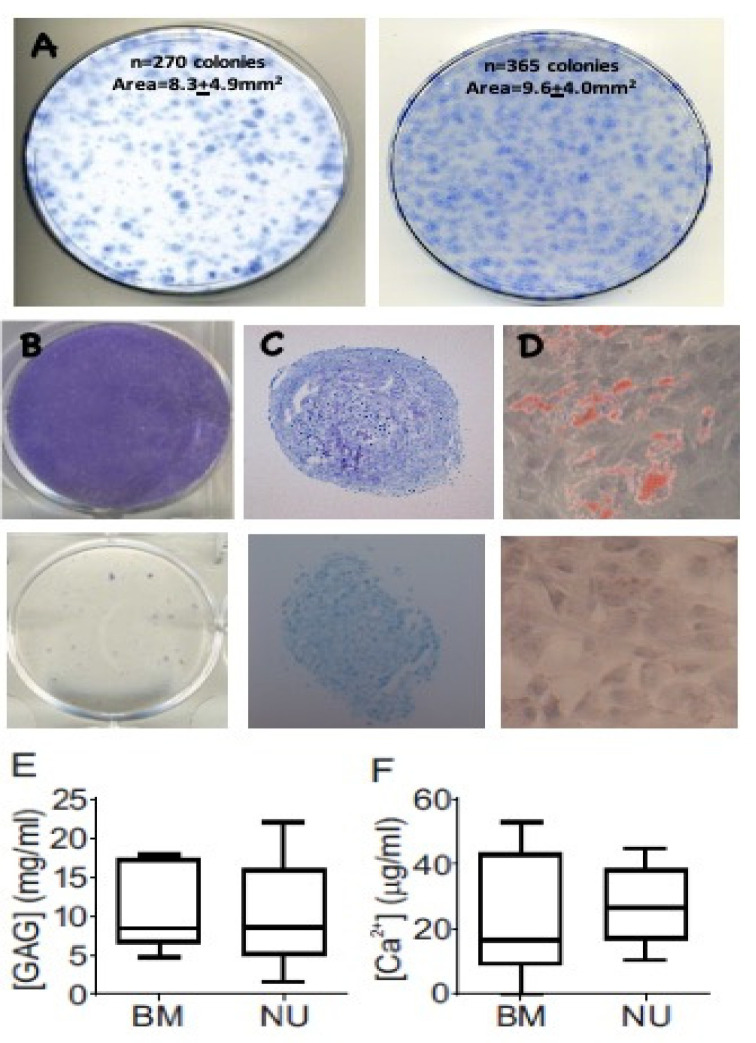
Colony formation (**A**) and multi-lineage differentiation potential (**B**–**E**) of non-union (NU)-derived mesenchymal stromal cells (MSCs). Enzymatically digested non-union tissue readily forms similar-sized colonies in vitro (**A**, left panel) compared with control MSCs derived from bone marrow (BM) aspirates (**A**, right panel). Culture expanded adherent cells undergo osteogenic differentiation, as shown by alkaline phosphatase staining (**B**); chondrogenic differentiation, as shown by toluidine blue staining of chondrogenic pellets (**C**); and adipogenic differentiation, as shown by oil red staining (**D**). The bottom panels on **B**–**D** show cells stained after culturing in normal growth media. Quantitative measurements of chondrogenesis, measured by glycosaminoglycan (GAG) accumulation (**E**), and osteogenesis, measured by accumulation of Ca^2+^ (**F**), were comparable between BM MSCs (BM) (*n* = 6) and non-union derived MSCs (NU) (*n* = 6).

To investigate if NU tissue contained MSCs, CFU-F assays were undertaken on NU tissue digests and their trilineage differentiation capacity was compared with BM MSCs that are commonly used as the standard control for the assessment of MSC colony-forming ability and multipotentiality (Figure 1). Following enzymatic digestion and in vitro cultivation, cells isolated from NU tissue readily formed colonies (Figure 1A, left panel) similar to MSCs from BM aspirates (Figure 1A, right panel). In vitro expansion of colony forming cells followed by induction of differentiation in osteogenic, chondrogenic, and adipogenic media showed that cells from NU tissue were capable of osteogenic, chondrogenic, and adipogenic differentiation, as shown by positive alkaline phosphatase staining, toluidine blue, and oil red O staining, respectively (Figure 1B–D, top panels), compared with NU-derived cells grown in normal growth media (Figure 1B–D, bottom panels). Quantitative measurement of chondrogenesis by glycosaminoglycan (GAG) accumulation showed parity between NU- and BM-derived MSCs (Figure 1E), as did quantitative measurement of osteogenesis by calcium accumulation (Figure 1F). These data indicated that NU tissue had cells with robust in vitro MSC activity.

### 3.2. Cellular Composition of NU Tissue

**Figure 2:**
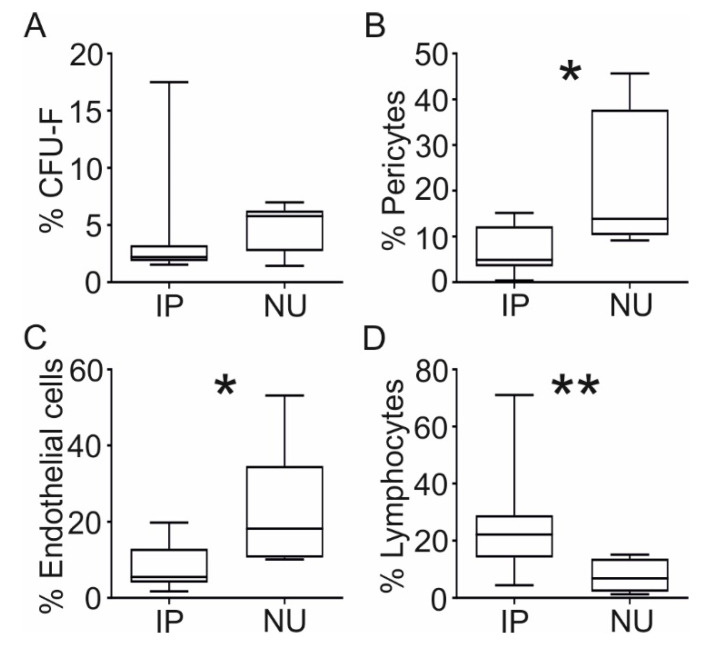
A comparison of cellular composition between NU and induced periosteum (IP) tissue. Comparison of the percentage of colony forming cells assessed by colony forming unit fibroblast (CFU-F) assay following enzymatic digestion and in vitro culture in induced periosteum (IP) (*n* = 8) and non-union (NU) tissue (*n* = 6) (**A**). Flow cytometry analysis of the percentage of cells expressing cell surface markers consistent with pericytes (CD45^−^CD34^−^CD146^+^) (**B**), endothelial cells (CD45^−^CD31^+^) (**C**), and lymphocytes (CD45^+^side scatter low) (**D**), following enzymatic digestion of IP (*n* = 10) and NU tissue (*n* = 6). * denotes *p* < 0.05, ** denotes *p* < 0.01.

To investigate the frequency of MSCs, as well as other cells important in the bone repair process in NU tissue, CFU-F assay and flow cytometry using NU tissue digests were performed, and the data were compared to IP tissue, which is known to be an active reservoir of such cells [38]. IP was chosen as the most appropriate positive control in these experiments because, similarly to NU tissue, it is newly-formed and vascularised in vivo during the regenerative process of fracture healing. Resident MSCs were measured using CFU-F assay and flow cytometry for cells expressing phenotypic markers consistent with pericytes [38]. Colony forming potential of NU and IP tissue measured by CFU-F assay was comparable (Figure 2A), indicating no significant difference in MSC content between these tissues. However, the NU tissue contained a significantly greater percentage of cells expressing phenotypic markers consistent with pericytes: NU had a median of 13.8% pericytes (range 9.2–45.7) compared with 4.9% in IP tissue (range 0.3–15.1, *p* = 0.036, Figure 2B), indicating a possible accumulation of pericytes, but not MSCs in the NU tissue. NU tissue also contained a greater percentage of CD31^+^ endothelial cells; NU had a median proportion of 18.2% endothelial cells (range 10.1–53.1) compared with 5.5% in IP (range 1.7–19.8, *p* = 0.016, Figure 2C). Finally, NU tissue had a reduced number of lymphocytes; NU had a median proportion of 6.8% lymphocytes (range 1.3–15.1) compared with 22.2% (range 4.4–71.1, *p* = 0.007) in IP (Figure 2D). Supplementary Figure S2E demonstrates the original flow cytometry plots for these cell populations. These data provided a first indication for the disturbed MSC–pericyte relationship in the NU tissue.

### 3.3. Histological Comparison of NU and IP Tissue

**Figure 3:**
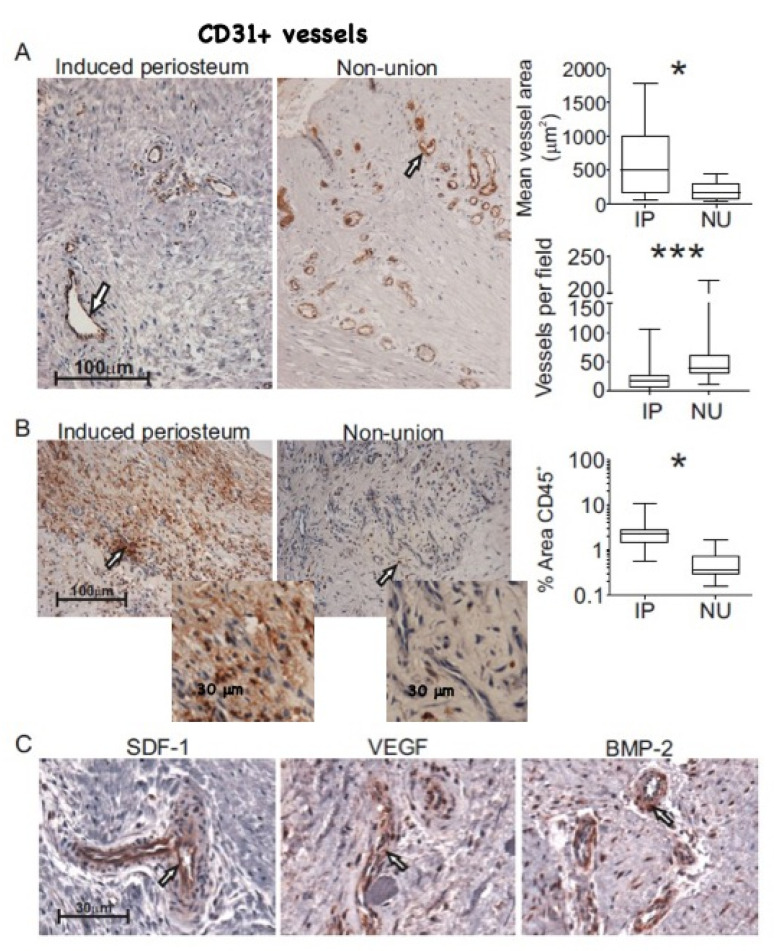
Histological examination of NU tissue. Comparison of blood vessel size highlighted by immunohistochemistry (IHC) staining for CD31 expression in induced periosteum (IP) and non-union (NU) tissue, representative photomicrographs (200× magnification, scale bar 100µm) showing blood vessels (left and middle panels), comparison of mean internal vessel area by manual blind scoring (top right), and comparison of mean number of blood vessels per field (bottom right) (**A**). Comparison of leukocyte content highlighted by IHC staining for CD45 expression in IP and NU tissue, representative photomicrographs (200× magnification, scale bar 100 µm) showing leukocyte content (left and middle panels), and automated comparison showing the average area of positive staining per field (right) (**B**). Higher magnification images of CD45+ cells in IP and NU tissue are shown in the inserts. IHC staining highlighting perivascular location of stromal-derived factor-1 (SDF-1), vascular endothelial growth factor (VEGF), and bone morphogenetic protein-2 (BMP-2) in NU tissue (scale bar 30 µm) (**C**). All data IP (*n* = 8) and NU tissue (*n* = 9), arrows show regions of positive staining (brown). * denotes *p* < 0.05, *** denotes *p* ≤ 0.001.

To investigate whether disparities in the observed numbers of pericytes, MSCs, and endothelial cells were related to different abundance of blood vessels in these tissues, IHC for CD31 was performed. By blinded manual measurement of blood vessel internal area, IP was found to contain a greater number of larger calibre blood vessels in comparison with NU tissue (Figure 3A), where we also noted the presence of several clusters of small calibre vessels. This observation was confirmed by blinded manual measurement of blood vessel internal area; median vessel area per sample in IP tissue was approximately 2.9-fold greater than that of NU tissue (500 µm and 174 µm, respectively, *p* = 0.046, Figure 3A). Alternative analysis by comparison of individual vessel size for each tissue type was highly significant (Supplementary Figure S1A) and analysis of vessel density showed a 2.4-fold increase in the number of vessels per field in NU compared with IP tissue (*p* = 0.001, Figure 3A). Together, these results and the abundance of endothelial cells measured by flow cytometry suggest differences in both blood vessel size and number. Moreover, consistent with flow cytometry data, CD45 staining for the presence of leukocytes showed a far greater abundance of tissue-resident leukocytes throughout IP in comparison with NU tissue (Figure 3B). This was confirmed by automated quantitative scoring of the proportion of area occupied by CD45 positively stained cells. The median CD45 positive area in IP was 2.2% (range 0.56–10.06) compared with 0.4% (range 0.15–1.68) in NU tissue (*p* = 0.005, Figure 3B). This was consistent with our flow cytometry data showing higher percentages of CD45+ lymphocytes in IP tissue compared with NU tissue, as shown on Figure 2D. Immunohistochemical analysis of NU tissue confirmed the presence of SDF-1, VEGF, and BMP-2 and their localisation to blood vessels (Figure 3C); this was consistent with our previous findings in IP tissue [38]. Haematoxylin and Eosin staining of NU tissue showed the presence of small fragments of dead bone, shown by their lack of viable osteocytes (Supplementary Figure S1B), suggesting inadequate clearance by osteoclasts.

### 3.4. Transcriptional Profile Comparison of IP and NU Derived MSCs

**Figure 4:**
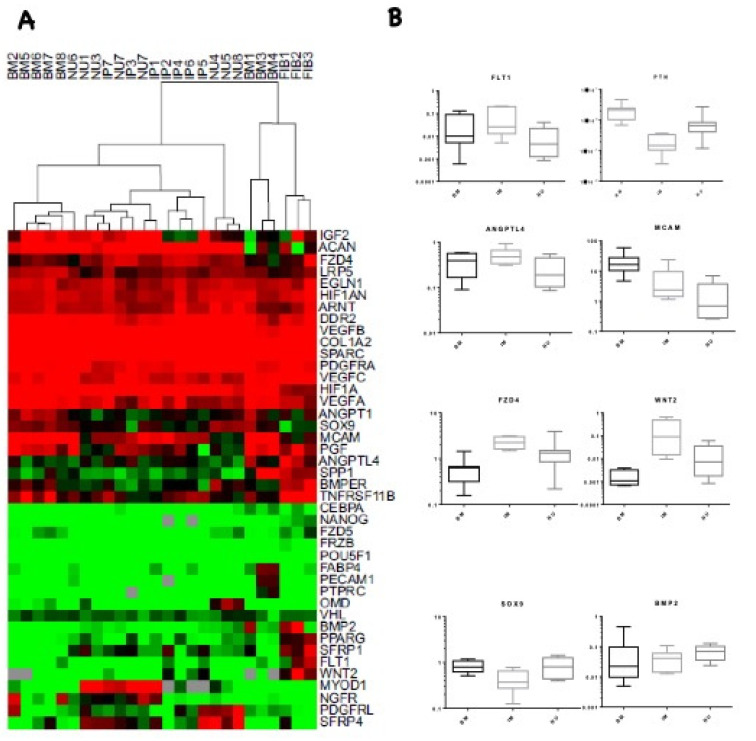
Differentially expressed transcripts in MSCs derived from IP and NU tissue. Cluster analysis of log 2 transformed relative expression data: 2−ΔCt normalised to HPRT. IP—induced periosteum (*n* = 7), NU—non-union (*n* = 8), BM—bone marrow (*n* = 8), and Fib—skin fibroblasts negative control (*n* = 3). Green < HPRT, red > HPRT, black = HPRT, and grey is missing data. Genes were analysed when 80% present, complete linkage clustering was performed and viewed using Java Treeview (**A**). Genes showing marked differences in relative expression between NU MSCs and IP MSCs; BM MSCs included as controls. Genes involved in regulation of endothelial cells (*FLT1, PTN, ANGPL4*, and *MCAM*), Wnt signalling genes (*FZD4* and *WNT2*), and genes involved in MSC differentiation (*SOX9* and *BMP-2*) (**B**). All data IP (*n* = 7), NU (*n* = 8), BM (*n* = 8). R.E.U.—relative expression unit.

The combined colony forming potential, IHC, and flow cytometry data indicated that, while MSCs were abundant in NU tissue, the proportion of endothelial cells, pericytes, and lymphocytes, as well as blood vessel diameters and density, differed. As MSCs are known to participate in tissue formation and remodelling via their trophic functions of chemo-attraction and angiogenic support activity [16,17,18,19], we investigated the expression of possible mediators of these activities, using a 48-gene array encompassing genes involved in tissue formation and angiogenesis. This was performed on NU MSCs and compared with BM MSCs (positive control), skin fibroblasts (negative control), and IP MSCs, with the latter representing MSCs from a highly-osteogenic environment (Figure 4). Cluster analysis of 48-gene TLDA data showed tight clustering of fibroblasts away from IP, NU, and most BM-derived MSCs. BM MSCs flanked IP and NU MSC profiles, while IP and NU MSCs clustered together with no obvious separation from each other (Figure 4A).

Comparison of the expression of individual transcripts between IP- and NU-derived MSCs and control BM MSCs showed interesting trends. Among genes with predominantly endothelial regulatory roles, expression of *FLT1* and *ANGPTL4* was lower in NU tissue compared with both BM MSCs and IP MSCs. These differences were statistically significant when NU MSCs were compared with IP MSCs (*p* = 0.021 and *p* = 0.012 respectively, Mann–Whitney U test). *MCAM1* expression was similarly higher in NU MSCs compared with IP MSCs, although short of significance (*p* = 0.059), whereas *PTN* was significantly increased in NU (*p* = 0.003, Mann–Whitney U test) compared with IP tissue (Figure 4B). Expression of Wnt pathway genes *FZD4* and *WNT2* was decreased in NU MSCs compared with IP MSCs (*p* = 0.016 and 0.028 respectively, Mann–Whitney U test) (Figure 4B). We did not observe any significant difference in other Wnt related genes, including *DKK1, DKK2*, *SOST*, and *KREMEN1*. Expression of *SOX9* and *BMP-2* both involved in MSC differentiation were increased in NU tissue MSC compared with IP MSCs; however, this only reached significance in the case of *SOX9* (*p* = 0.012, Mann-Whitney U test) (Figure 4B).

### 3.5. Assessment of MSC Mediated Angiogenesis

**Figure 5:**
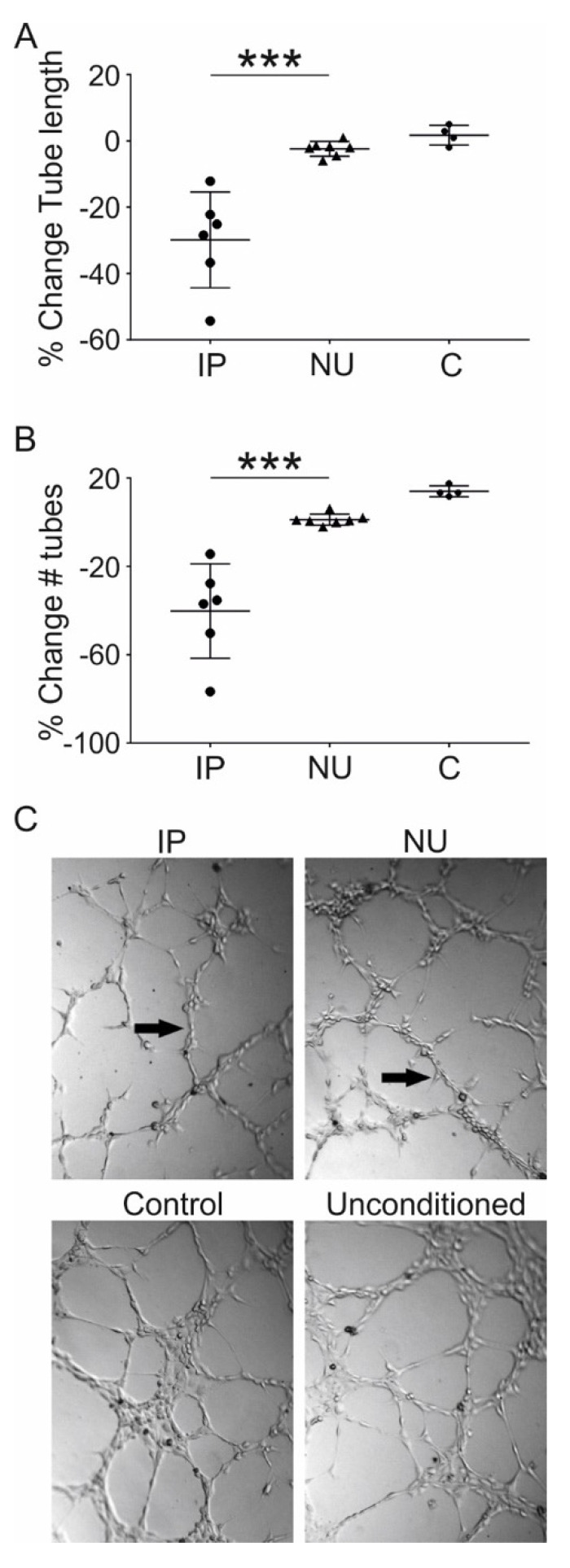
MSC from induced periosteum inhibit early in-vitro angiogenesis. Angiogenesis measured by total tube length (**A**) and total number of tubes (**B**) shows a significant inhibitory effect of media conditioned by MSCs originating from induced periosteum (IP) compared with non-union (NU) and positive control human umbilical vein endothelial cell (HUVEC) endothelial cell growth media (**C**). Values are normalised to unconditioned MSC growth media and the number of MSCs is counted at the end of the conditioning period. Photomicrographs showing representative examples of tube formation in induced periosteum, non-union, HUVEC control, and unconditioned media treated wells (**C**). All data IP (*n* = 6), NU (*n* = 7), C (four technical replicates). *** denotes *p* ≤ 0.001.

To understand the functional effect of differences in transcriptional expression between MSCs originating from IP and NU tissue, a matrigel-based angiotube formation assay that reflects the early stages of angiogenesis was performed [51,52], using conditioned IP- and NU-MSC growth media. Some angiogenesis was observed using unconditioned MSC growth media; therefore, this was used as a baseline and the contribution of MSC conditioning was standardised against this. There was striking inhibition of in vitro angiogenesis in media conditioned with IP origin MSCs compared with NU origin MSCs. Several parameters were assessed: the total tube length (Figure 5A) and the total number of tubes formed (Figure 5B). In all cases, conditioned media from IP MSCs had a significant negative effect on early angiogenesis compared with NU-derived MSCs (all *p* = 0.001). These effects are illustrated by photomicrographs of endothelial tube formation taken at the end-point of this assay (Figure 5C).

## 4. Discussion

This study examined the MSC functional capacity, cellular content, histology, and vascularity of tissue harvested from the site of atrophic NUs. Initially, we investigated the possibility that NU tissue either lacked or had very low MSCs content. Our results show that this is not the case, in agreement with previously published findings in this regard [26], and show that, following digestion, the MSC frequency in NU tissue is substantially greater than that in BM aspirates [39] and comparable to IP [38], as well as to enzymatically digested cancellous bone [46]. The possibility that MSCs isolated from NU tissue were deficient in their ability to differentiate was then investigated. Contrary to previously published data [26], we found no significant difference between NU- and BM-derived MSCs with regards to in vitro differentiation capacity. Together, these results suggest that NU tissue contains a large number of MSCs with no inherent defect in differentiation capacity in vitro.

Examination of the total cellular content of NU in comparison with IP tissue using flow cytometry highlighted several important differences; NU tissue contains a greater proportion of cells expressing markers specific to pericytes and endothelial cells and fewer lymphocytes. Although some caution should be applied to analysis of cellular content based exclusively on measures of overall relative percentages, this nevertheless suggests differences in the vascularity between these tissues, which were confirmed in vivo. Histological examination revealed that, even though there are no differences in regard to expression of key perivascular proteins, as their localisation in NU tissue closely replicated that previously observed in IP tissue [38], NU tissue contained on average a greater number of much smaller diameter blood vessels compared with IP tissue, which may have important implications for the supply of oxygen and nutrients in these tissues. Histological examination also revealed striking differences in the leukocytic content of NU and IP tissues leukocytes, which were largely absent in NU. This confirms our flow cytometry findings and, furthermore, suggests tissue infiltration rather than localisation within blood vessels as the cause of the observed higher number of lymphocytes in IP tissue. One may, however, argue that the presence of lymphocytes in the IP could be secondary to the foreign body reaction mounted against the Poly(methyl methacrylate)(PMMA) spacer, rather than an increased permeability. While this is a possibility, because of the fact that the IP tissue was harvested on average seven weeks after the cement implantation, at that stage, it is unlikely that an inflammatory reaction was still active. It may well be that both the foreign body reaction and an increased permeability contributed to this finding. Together, these results point to dissimilarities in blood vessels number and size as key differences between these IP and NU tissues.

In order to ascertain what role, if any, MSCs may play in causing the differences in vascularity observed in these tissues, we examined the transcriptional profile of MSCs derived from NU and IP. Individual analysis of gene expression highlighted considerable differences in the expression of several genes. The expression of *FLT1,* the gene encoding Fms-related tyrosine kinase 1, also known as *VEGFR-1*, was significantly reduced in NU compared with IP tissue. *FLT1* is a member of the vascular endothelial growth factor receptor family [53,54] and exists as a soluble and membrane bound form. *FLT1* expression in periosteum-derived cells has been linked to osteogenesis [55], but is also widely recognised for its role in angiogenesis. Homozygous deletion of flt-1 in mice is embryonically lethal owing to uncontrolled accelerated endothelial cell differentiation, leading to the formation of disorganised and ineffective vasculature [56]. Similarly, loss of flt-1 in developing vessels in vitro results in an increased tip cell formation and sprout initiations, but a significant reduction in the number of new stable conduits [57]. These effects were echoed in our study, where we observed a greater proportion of endothelial cells, but a significant decrease in vessel diameter NU tissue, which may indicate relative vessel immaturity.

Interestingly, expression of the gene encoding pleiotrophin (*PTN*) was significantly greater in NU-derived MSCs. *PTN* is a secreted heparin-binding growth factor that may play a significant role in bone remodelling either directly by influencing MSCs, or indirectly by influencing angiogenesis [58]. Increased angiogenesis, modulation of the vascular morphology, and deranged vessel perfusion and functionality have been recently described in gliomas over-expressing *PTN* [59], in line with our data related to both vessel number and size, and different degrees of leukocyte infiltration in NU tissue compared with IP tissue. Overall, our gene expression data are in line with previous studies showing that fracture healing is impaired in adult PTN-over-expressing mice [60], perhaps as a result of antagonizing the effect of *BMP-2* [60,61].

Expression of the gene encoding angiopoietin-like 4 (*ANGPTL4*) was reduced in NU-derived MSCs. *ANGPTL4* promotes remodelling of the cartilage matrix during chondrogenic differentiation of MSCs [62]; because expression of *ANGPTL4* decreased in NU tissue-derived MSCs, this could indicate defective endochondral ossification. Another immerging role of *ANGPTL4* is in regulation of vascular permeability [63,64,65]; this is interesting given the clear difference in leukocyte infiltration observed between NU and IP tissues. Lack of leukocyte infiltration to NU tissue, specifically osteoclasts, may result in decreased clearance of dead bone fragments and have significant implications for disease pathogenesis.

Expression of the genes encoding *WNT2* (wingless-type MMTV integration site family, member 2), a member of the canonical Wnt signalling pathway, as well as *FZD4* (frizzled class receptor 4) and LRP5 (lipoprotein-related protein 5), which encode a receptor and co-receptor of the Wnt/β-catenin signalling pathway, respectively, were reduced in NU tissue, although not significantly in the case of *LRP5*. It is widely accepted that an increase in *LRP5*/6-mediated signalling leads to an increase in bone mass [66,67,68]. Therefore, it is possible that the in vitro assays used in this study to assess osteogenesis lack the subtlety to reflect changes to MSC Wnt signalling that in vivo would make a substantial difference to bone formation. The increased level of *SOX9* transcript, an important transcription factor in chondrogenesis [69], supports this assertion. Differences in osteogenic culture conditions may also explain the disparity between this study and the findings of Bajada et al., who observed loss of osteogenic potential in MSCs originating from NU tissue [26].

Finally, a tube formation assay was performed to understand the functional consequences of altered expression of MSC secreted factors associated with angiogenesis. Although media conditioned with MSCs from NU tissue had a negligible effect on the early stages of angiogenesis, as measured by this assay, MSCs conditioned media from IP tissue caused a striking reduction in angiogenesis. One possible explanation for this could be that growth factors responsible for the relatively high baseline angiogenesis seen in the unconditioned media were depleted in IP conditioned media. However, in general, fewer MSCs were recorded at the end of the conditioning phase in IP compared with NU, making this explanation unlikely. This leaves the possibility that MSCs from IP are actively secreting factors that inhibit early vessel formation in this assay. This is consistent with our histology observations that the number of both endothelial cells and pericytes were increased in NU tissue, compared with IP tissue, but blood vessel diameters were significantly reduced in NU tissue compared to IP tissue. Given that vascular flow is related to the fourth power of the vessel radius according to Poiseuille’ equation our findings indicate substantial differences in vascular flow between IP and non-union tissue, which will be manifold higher in the IP tissue. 

These data appear to run contrary to the well-established idea that angiogenesis promotes bone healing and that MSCs stimulate angiogenesis [19,20,21]. However, despite its inhibitory effect on the early stages of angiogenesis, *FLT-1* is integral to vessel stabilisation and spatial arrangement [70]. Therefore, we suggest that MSCs in IP tissue may be involved in guiding this process and that NU MSCs either lack this ability or have not received the correct stimulus to initiate it. Regardless, the fact that this phenotype persists in vitro for several rounds of culture expansion has wider implications in the field of MSC therapy and suggests that careful consideration should be given to the angiogenic phenotype of therapeutic MSCs. For example, MSCs exhibiting the phenotype of IP MSCs are unlikely to be beneficial for therapies requiring rapid vascularisation of an avascular scaffold. Equally, in the treatment of NU, MSCs that are able to stabilise an already existing vascular network and improve blood flow would be beneficial. Additionally, understanding of the stimuli needed to switch MSCs between these two phenotypes, particularly hypoxia, may have important implications for both regenerative medicine and cancer therapeutics, where MSCs are heavily implicated in driving tumour angiogenesis [71,72,73].

One of the limitations of this study is its descriptive component. However, angiogenesis was demonstrated at protein and functional levels in addition to the data obtained by qPCR. Moreover, the samples harvested and analysed from patients represent a unique feature and contribute further to the growing literature of non-union pathogenesis and IP research. They highlight the importance of a cross-talk between local MSCs and endothelial cells in the formation and maturation of a functional vascular network in the regenerating tissue. Future work would be desirable and should test the candidate molecules using an animal model in order to further dissect the role of each individual molecular component in the development of atrophic non-unions.

## Supplementary Materials

The following are available online at https://www.mdpi.com/2077-0383/9/6/1628/s1, Figure S1: Additional histological comparison; Figure S2E: Cell populations in tissue digests of non-union tissue; Table S1: Antibodies, Table S2: Taqman assays used for gene expression study; Table S3: Control tissues used throughout the study.
