# Supplementary material for: Regulation of Angiogenesis Discriminates Tissue Resident MSCs from Effective and Defective Osteogenic Environments
Source: J Clin Med. 2020 May 28;9(6):1628. doi: 10.3390/jcm9061628 (PMC7355658; doi:10.3390/jcm9061628)
Supplement: Supplementary file 1 [file jcm-09-01628-s001.zip › jcm-736513-supplementary/Supplementary table 3_NU_R2.docx]

**Supplementary Table 3. Control tissues used throughout the study**

| Colony formation and differentiation capacity of NU MSCs | Bone marrow aspirate |
| --- | --- |
| Cellular composition of NU tissue | Induced periosteum tissue |
| Histology and flow cytometry | Induced periosteum tissue |
| Transcriptional profiling | Induced periosteum tissue, bone marrow aspirate, skin fibroblasts (negative control) |
| Assessment of angiogenesis | Induced periosteum tissue |
