# Supplementary material for: Regulation of Angiogenesis Discriminates Tissue Resident MSCs from Effective and Defective Osteogenic Environments
Source: J Clin Med. 2020 May 28;9(6):1628. doi: 10.3390/jcm9061628 (PMC7355658; doi:10.3390/jcm9061628)
Supplement: Supplementary file 1 [file jcm-09-01628-s001.zip › jcm-736513-supplementary/Supplementary table 2.docx]

| ***Gene*** | **Taqman assay** | **Description** |
| --- | --- | --- |
| *18s* | Hs 99999901_s1 | 18S ribosomal RNA |
| *ACAN* | Hs 00153936_m1 | aggrecan |
| *ANGPT1* | Hs 00181613_m1 | angiopoietin 1 |
| *ANGPTL4* | Hs 01101127_m1 | angiopoietin-like 4 |
| *ARNT* | Hs 01121918_m1 | aryl hydrocarbon receptor nuclear translocator |
| *BMP2* | Hs 00154192_m1 | bone morphogenetic protein 2 |
| *BMP7* | Hs 00233477_m1 | bone morphogenetic protein 7 |
| *BMPER* | Hs 00403062_m1 | BMP binding endothelial regulator |
| *CEBPA* | Hs 00269972_s1 | CCAAT/enhancer binding protein (C/EBP) |
| *COL1A2* | Hs 01028971_m1 | collagen, type I, α2 |
| *DDR2* | Hs 00178815_m1 | discoidin domain receptor tyrosine kinase 2 |
| *EGLN1* | Hs 00254392_m1 | egl nine homolog 1 (*C. elegans*) |
| *FABP4* | Hs 00609791_m1 | fatty acid binding protein 4, adipocyte |
| *FLT1* | Hs 01052937_m1 | fms-related tyrosine kinase 1 (VEGFR1) |
| *FRZB* | Hs 00173503_m1 | frizzled-related protein |
| *FZD4* | Hs 00201853_m1 | frizzled family receptor 4 |
| *FZD5* | Hs 00258278_s1 | frizzled family receptor 5 |
| *GAPDH* | Hs 99999905_m1 | glyceraldehyde-3-phosphate dehydrogenase |
| *HIF1A* | Hs 00936371_m1 | hypoxia inducible factor 1, α subunit |
| *HIF1AN* | Hs 00215495_m1 | hypoxia inducible factor 1, α subunit inhibitor |
| *HPRT* | Hs 99999909_m1 | hypoxanthine phosphoribosyltransferase 1 |
| *IGF2* | Hs 01005963_m1 | insulin-like growth factor 2 |
| *KDR* | Hs 00911702_m1 | kinase insert domain receptor (VEGFR2) |
| *LRP5* | Hs 00182031_m1 | low density lipoprotein receptor related protein 5 |
| *MCAM* | Hs 00174838_m1 | melanoma cell adhesion molecule |
| *MYOD1* | Hs 00159528_m1 | myogenic differentiation 1 |
| *NANOG* | Hs 02387400_g1 | Nanog homeobox |
| *NGFR* | Hs 00182120_m1 | nerve growth factor receptor |
| *OMD* | Hs 00192325_m1 | osteomodulin |
| *PDGFRA* | Hs 00998018_m1 | platelet-derived growth factor receptor α |
| *PDGFRL* | Hs 00185122_m1 | platelet-derived growth factor receptor-like |
| *PECAM1* | Hs 00169777_m1 | platelet/endothelial cell adhesion molecule |
| *PGF* | Hs 01119262_m1 | placental growth factor |
| *POU5F1* | Hs 00999632_g1 | POU class 5 homeobox 1 |
| *PPARG* | Hs 01115513_m1 | peroxisome proliferator-activated receptor γ |
| *PTN** | Hs 00383255_m1 | pleiotrophin |
| *PTPRC* | Hs 00894732_m1 | protein tyrosine phosphatase, receptor C |
| *PTPRZ1** | Hs 00960146_m1 | protein tyrosine phosphatase, receptor type Z1 |
| *SFRP1* | Hs 00610060_m1 | secreted frizzled-related protein 1 |
| *SFRP4* | Hs 00180066_m1 | secreted frizzled-related protein 4 |
| *SOX9* | Hs 00165814_m1 | SRY (sex determining region Y)-box 9 |
| *SPARC* | Hs 00277762_m1 | osteonectin |
| *SPP1* | Hs 00959010_m1 | secreted phosphoprotein 1 |
| *TNFRSF11B* | Hs 00900360_m1 | tumor necrosis factor receptor superfamily 11b |
| *VEGFA* | Hs 00900058_m1 | vascular endothelial growth factor A |
| *VEGFB* | Hs 00173634_m1 | vascular endothelial growth factor B |
| *VEGFC* | Hs 01099206_m1 | vascular endothelial growth factor C |
| *VHL* | Hs 01650959_m1 | von Hippel-Lindau tumor suppressor |
| *WIF1* | Hs 00183662_m1 | WNT inhibitory factor 1 |
| *WNT2* | Hs00608224_m1 | wingless-type MMTV integration site family 2 |
|  |  |  |
|  |  |  |
|  |  |  |

Supplementary table 2: Taqman assays used for gene expression study
