# Supplementary material for: Regulation of Angiogenesis Discriminates Tissue Resident MSCs from Effective and Defective Osteogenic Environments
Source: J Clin Med. 2020 May 28;9(6):1628. doi: 10.3390/jcm9061628 (PMC7355658; doi:10.3390/jcm9061628)
Supplement: Supplementary file 1 [file jcm-09-01628-s001.zip › jcm-736513-supplementary/Suppl Figure 2E.docx]

**Figure 2E**


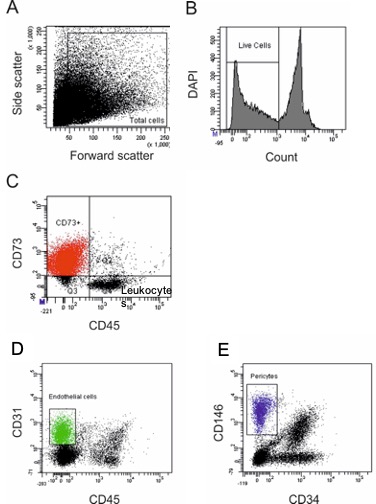


Figure 2E: Cell populations in tissue digests of non-union tissue.

Cells are isolated from debris based on forward and side scatter profile (A). Dead cells are removed based on uptake of DAPI (B). Leukocytes are identified by positive expression of CD45 and negative expression of CD73 (C). Endothelial cells are identified based on negative expression of CD45 and positive expression of CD31 (CD45^-^CD31^+^) (D). Pericytes are identified by pre-gaiting on negative expression of CD45 then by negative expression of CD34 and positive expression of CD146 (CD45^-^CD34^-^CD146^+^) (E).
