# Supplementary material for: Regulation of Angiogenesis Discriminates Tissue Resident MSCs from Effective and Defective Osteogenic Environments
Source: J Clin Med. 2020 May 28;9(6):1628. doi: 10.3390/jcm9061628 (PMC7355658; doi:10.3390/jcm9061628)
Supplement: Supplementary file 1 [file jcm-09-01628-s001.zip › jcm-736513-supplementary/Supplementary table 1.docx]

| Specificity | Application | Conjugate | Clone | Host | Dilution | Manufacturer |
| --- | --- | --- | --- | --- | --- | --- |
| CD31 | IHC | None | JC70A | Mouse | 1:50 | DakoCytomation |
| CD31 | FC | BV605 | WM59 | Mouse | 1:20 | Biolegend |
| CD45 | IHC | None | 2B11+PD7/26 | Mouse | 1:50 | DakoCytomation |
| CD45 | FC | PE-Cy7 | HI30 | Mouse | 1:20 | BD Pharmingen |
| CD146 | FC | FITC | P1H12 | Mouse | 1:20 | BD Pharmingen |
| BMP-2 | IHC | None | Polyclonal | Rabbit | 1:100 | AbD Serotech |
| SDF-1 | IHC | None | 79018 | Mouse | 1:20 | R&D Systems |
| VEGF | IHC | None | Polyclonal | Rabbit | 1:20 | Santa Cruz Biotech |

Supplementary table 1: Antibodies
