# Supplementary figures and images for: Regulation of Angiogenesis Discriminates Tissue Resident MSCs from Effective and Defective Osteogenic Environments
Source: J Clin Med. 2020 May 28;9(6):1628. doi: 10.3390/jcm9061628 (PMC7355658; doi:10.3390/jcm9061628)

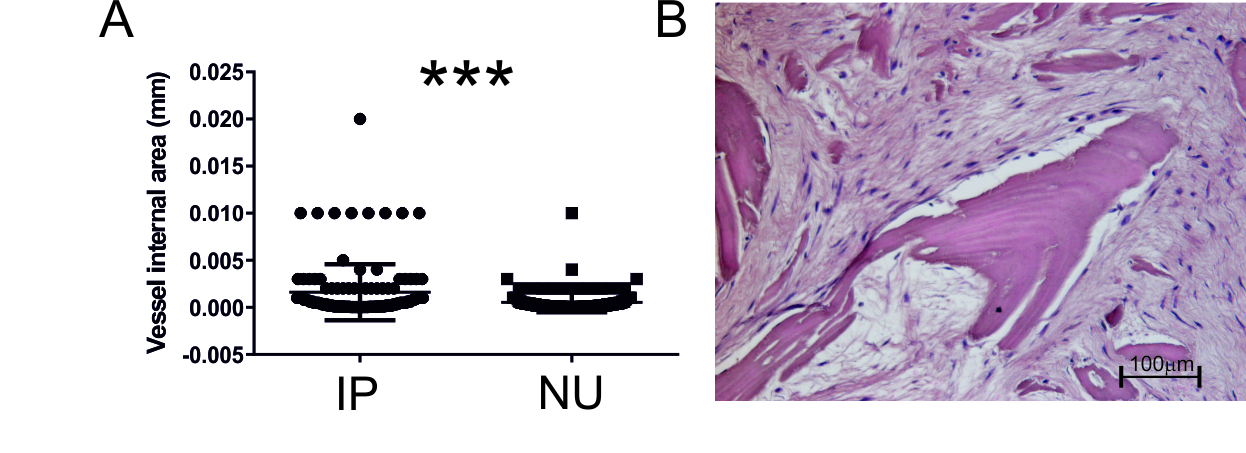

Supplement: Supplementary file 1 [file jcm-09-01628-s001.zip › jcm-736513-supplementary/Suppl Figure 1. jpg.jpg]
